# Corrosion protection properties of different inhibitors containing PEO/LDHs composite coating on magnesium alloy AZ31

**DOI:** 10.1038/s41598-021-81029-6

**Published:** 2021-02-02

**Authors:** Gen Zhang, E Jiang, Liang Wu, Weigang Ma, Hong Yang, Aitao Tang, Fusheng Pan

**Affiliations:** 1 Nuclear Power Institute of China, Chengdu, 610213 Sichuan China; 2 College of Materials Science and Engineering, Chongqing University, Chongqing, 400044 China; 3 National Engineering Research Center for Magnesium Alloys, Chongqing University, Chongqing, 400044 China

**Keywords:** Nanoscale materials, Chemistry

## Abstract

Corrosion inhibitors 2,5-pyridinedicarboxilate (PDC), sodium metavanadate (SMV) and 5-aminosalicylate (AS) were impregnated into porous PEO coatings respectively via vacuuming process, followed by fast sealing treatment in a Ce containing solution. After that layered double hydroxides (LDHs) based nanocontainers were respectively prepared on them via hydrothermal treatment. In frame of this work it was shown, that sealing effect for the pore was provided by formation of new phase CeO_2_ on the surface of PEO coatings. And, hydrothermal preparation for preparing LDHs leaded obvious changes in structure and thickness of the coatings. In addition, impregnation of inhibitors was in favor of improving LDHs content in final composite coatings. EIS result indicated that AS/Ce-HT specimen exhibited a best corrosion protection.

## Introduction

It is well known that magnesium (Mg) has highest chemical activity (the standard equilibrium potential of Mg/Mg^2+^ is − 2.4 V_NHE_) compared with other engineering metals, such as Fe (− 0.409 V_NHE_), Al (− 1.706 V_NHE_), which obviously limits its applications in a larger scale^1–3^. It is generally accepted that the Mg corrosion reactions can be written as^4^:

$$ {\text{Mg}} \to {\text{Mg}}^{{{2} + }} + {\text{2e}}^{ - } \;\;\; \left( {{\text{anodic}}\;{\text{reaction}}} \right) $$

$$ {\text{2H}}^{ + } + {\text{2e}}^{ - } \to {\text{H}}_{{2}} \uparrow \;{\text{or}}\; {\text{2H}}_{{2}} {\text{O}} + {\text{2e}}^{ - } \to {\text{H}}_{{2}} \uparrow + {\text{2OH}}^{ - } \;\;\;\left( {{\text{cathodic}}\;{\text{reaction}}} \right) $$

In fact, up to now, there is also dispute about the anodic reaction of Mg corrosion, such as Song et al.^5^ proposed a concept of anodic hydrogen evolution to describe hydrogen evolution associated with anodic dissolution. Due to the nonhomogeneity within Mg alloys, caused by the composition, microstructure and even crystal orientation, it will inevitably lead to micro-galvanic acceleration of corrosion, so that resulting in the local corrosion of Mg alloys. In this progress, the Mg matrix always (typically α-phase) acts as a micro-anode and is preferentially dissolved, cathodic hydrogen evolution occurs at the micro-cathode region acted by non-uniformly distributed alloying element solute, secondary phase or impurity particles (mainly Fe, Cu, Ni). As suggested Song et al.^2^, the corrosion potential difference between the anode and cathode is a critical variable determining the galvanic corrosion rate. In a word, in theory, there are several effective strategies to suppress Mg alloys’ corrosion: (i) insulation of electrical connection between the anode and cathode; (ii) inhibition of anodic Mg dissolution and (iii) inhibition of cathodic hydrogen evolution.

As for the first strategy, preparation of barrier coatings on Mg alloys can prevent underlying Mg substrate from in contacting with corrosion media, so that blocks the species exchange between the electrolyte and anodic/cathodic surface areas. So far, various physical and chemical coating including plasma electrolytic oxidation (PEO), anodizing, vapor deposition, chemical conversion coatings, organic coatings have been reported widely for Mg alloys^6–11^. However, in case of scratches, delamination or cracks, these damages can be the channel for the rapid intake of corrosive media and eventually lead to severe local corrosion.

**Figure 1:**
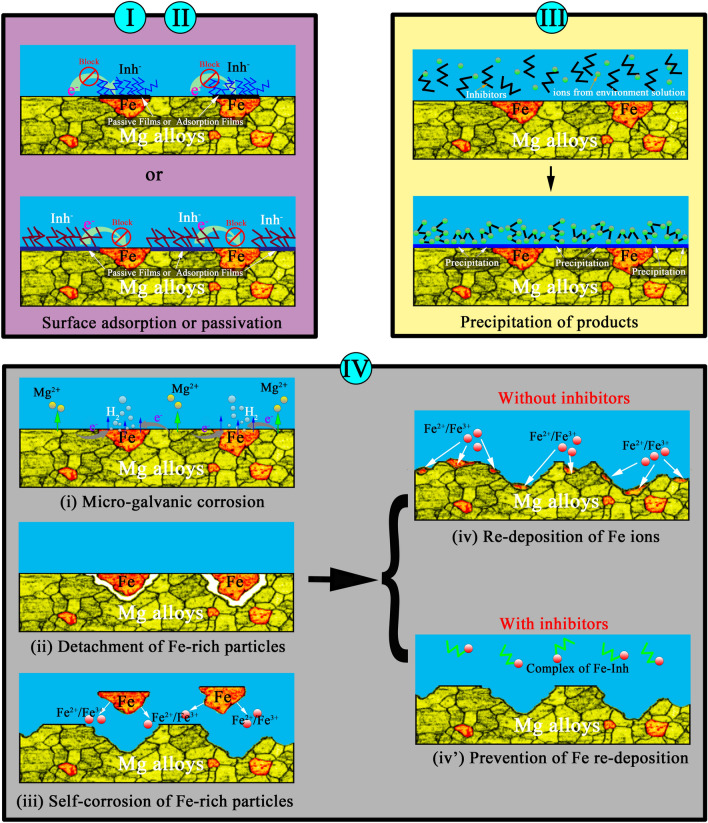
The schematic representation of inhibition mechanism of different kinds of corrosion inhibitors.

As for inhibition of the galvanic corrosion, application of inhibitors is one of the most a straightforward and effective methods of suppressing anodic reaction, cathodic reaction or both of them^12^. Different types of inorganic cations (Zn^2+^, Ce^3+^, La^3+^, etc.) and anions (fluorides, phosphates, silicate, etc.) have been reported as effective inhibitors to suppress corrosion of Mg alloys. Inhibiting effect of a number of organic inhibitors has also been investigated widely. For example, Frignani et al.^13^ used sodium salt of *N*-lauroylsarcosine, *N*-lauroyl-*N*-methyltaurine, dodecylbenzensulphonic acid or sodium lauryl sulphate as anionic surfactants. These surfactants adsorbed on Mg surface and resulted in a reduction in the cathodic activity, followed by formation of a precipitated layer with Mg^2+^, so that, the anodic Mg oxidation was further blocked. Lamaka et al.^14^ reported detachment of iron particles from the metallic substrate due to anodic Mg dissolution, and then self-corrosion of detached Fe-rich particles occurred with formation of Fe^2+^ and Fe^3+^. These species could be reduced to metallic Fe that leaded to deposition of Fe patches on Mg surface, which increased the total cathodic area. Hence, they proposed a concept of prevention of iron re-deposition for inhibiting Mg corrosion. Furthermore, the ability to suppress Mg dissolution of more than one hundred substances was screened comprehensively^15^. The inhibition mechanism of above mentioned organic molecules (shown in Fig. 1) is mainly ascribed to: (I) Surface adsorption, however, adsorptive efficiency is limited since the energy of the unoccupied 3*d* orbit of Mg is much higher, and resulting in a lower ability to accept π electrons from inhibitor molecules and lone-pair electrons from N, S and O atoms; (II) Surface passivation, i.e., formation of insoluble complex between Mg^2+^ or metallic ions of the second/tertiary phase. However, passive film is difficult form on Mg alloys, even highly reactive fluoride ions, since the affinity of Mg to react with species like O_2_, OH^-^ or H_2_ is high^15,16^; (III) Precipitation of products from the environment solution and (IV) Prevention of iron or noble metals re-deposition, in this theory, the ability of substances to form stable soluble complexes with Fe^2+^/Fe^3+^ was mainly considered.

Over the past decades, the emerging technology of smart coatings have been proposed and developed that has some functionality in addition to being a conventional barrier coating. Under this concept, known as self-healing or self-repair, such a coating has incorporated functionalities to sense damage, process the stimuli signals and to initiate single or multiple healing events or processes^17–20^. In order to obtain autonomous healing effect, one of strategy is by embedding extrinsic polymerizable healing agents in the coating. Another is by imbedding corrosion inhibitors as the healing agents^21^. When the coating is subjected to stimuli from the environment (such as pH, aggressive species, humidity or moisture), the corrosion inhibitor can leach into the coating defect and inhibit anodic or cathodic reaction, or both of them. The traditional manner of using inhibitors is to directly mix the inhibitor with the binder, but it will result in the poor dispersion and the interaction between the inhibitor and the polymer substrate. Encapsulation strategies to be as an effective solution has been proposed by White et al.^22^ and has been widely developed. Examples of encapsulating containers are: (i) inert capsules (e.g. mesoporous silica^23,24^, ceria^25,26^) which play no role other than to isolate the inhibitors from the polymeric material, and (ii) ion-exchange materials (e.g. clays^27,28^, zeolites^10,29^) which release the inhibitors in exchange for ions from environment^17,30^.

It had been shown that, the characteristic microstructure on PEO coatings has a great potential to serve as micro/nano reservoirs to store or carry inhibitor^31^. It is worth to mention that a post-sealing treatment is usually necessary step for avoiding loss of the loaded inhibitor from PEO coating. Some researchers^32–35^ have proposed many approaches to seal the pores of PEO coatings, i.e. sol–gel hybrid polymer.

Moreover, layered double hydroxides (LDHs, a kind of clay) as the nano-container have been widely investigated^36–41^, especially in combination with plasma electrolytic oxidation (PEO) coatings^42–46^. In our previous works, we reported an easy way to prepare MgAl-LDHs on anodic or PEO layer^47,48^, followed by confirming the two primary internal sources of Mg^2+^ and Al^3+^ cations for the formation of LDH coatings: degradation of the anodic or PEO layer and dissolution of the AZ31 substrate, and further a two-dimensional layer growth (2D) to three-dimensional (3D) growth model^49^ was proposed. Petrova et al.^50^ proposed to grow LDHs at ambient pressure on PEO treated magnesium alloy AZ91 in the presence of chelating agents. Recently a synergistic effect for active corrosion protection between Ce species and phosphate loaded LDHs was reported by us^30^. However, most of our study was focusing on loading inhibitors into LDHs, for improving active corrosion protection of coatings. Therefore, based on finding of Lamaka’s research^15^, 2,5-pyridinedicarboxilate (PDC), sodium metavanadate (SMV) and 5-aminosalicylate (AS) were chose to impregnate into porous PEO coatings respectively in this work. For avoiding loss of impregnated inhibitors during preparation process of LDHs, a fast sealing treatment was firstly performed in containing Ce solution. The reason for choosing Ce solution was that, presence of Ce ion in coatings was helpful to improve the active corrosion protection of coatings^30^. In such a way, impregnated inhibitors was firstly sealed by Ce conversion coating (Ce ions itself is used as a kind of inhibitors), and then fabrication of LDHs coating could enhance both the sealing effect and overall corrosion resistance of the composite coating. This final obtained composite coatings were characterized using SEM, and EDS, and then the corrosion protection were further investigated via electrochemical tests.

## Experimental methods

### Materials and reagents

The Mg alloy AZ31 with a nominal composition of Al 2.5–3.5%, Zn 0.6–1.3%, Mn 0.2–1%, Ca 0.04%, Si 0.1%, Cu 0.05% (all in wt%), and balance Mg. Deionized water was used as the solvent in this work.

### Specimens preparation

AZ31 alloy sheets with dimension of 20 mm × 20 mm × 5 mm were firstly ground on all surfaces up to 2000 grit using SiC papers, and then dried in warm air. The PEO processes were carried out at a constant voltage of 350 V for 600 s with duty ratio 20%, supplied by a pulse DC power source. The electrolyte containing 4 g/L NaAlO_2_ and 7.14 g/L NaOH was continuously stirred during the PEO treatment and kept at 20 ± 5 °C by a water cooling system.

Prior to impregnating the inhibitors into porous PEO layer, 0.1 M 2,5-pyridinedicarboxylic acid, sodium metavanadate and 5-aminosalicylic acid aqueous solutions with pH 7 (adjust with NaOH) were prepared respectively. The as-prepared PEO specimens were respectively immersed in the above three aqueous solutions in a well-sealed pressure container. A water pump was employed for constantly extracting the air from this enclosed container through a connected rubber hose. The inhibitors incorporating process lasted for 1 h. After incorporating process, the PEO specimens treated in the three different solutions were taken out of the container, and dried using warm air. The obtained specimens in this stage were designated as PDC, SMV and AS, respectively.

Subsequently, the pores in PEO layer of PDC, SMV and AS specimens were sealed in the aqueous solution containing 5 g/L Ce(NO_3_)_3_ and 0.5 g/L H_2_O_2_ at 90 °C for 10 min. After sealing treatment, the specimens were then dried using warm air. For clarity of discussion, the specimens obtained in this stage were called as PDC/Ce, SMV/Ce and AS/Ce, respectively.

For obtaining MgAl-LDH coating on PEO layer, as-prepared PDC/Ce, SMV/Ce and AS/Ce specimens were hydrothermally treated in 0.1 M NaNO_3_ solution in a Teflon-lined stainless steel autoclave at 398 K for 16 h, respectively. Eventually, the specimens were rinsed with distilled water, followed by drying. The final obtained specimens after hydrothermal treatment were designated as PDC/Ce-HT, SMV/Ce-HT and AS/Ce-HT, respectively.

### Characterization

Scanning electron microscopy (SEM; Tescan Vega3, Czech) equipped with an EDS detector was employed to investigate the surface and cross-sectional morphologies as well as the chemical compositions of the specimens. X-ray diffraction (XRD; Rigaku D/Max 2500X, Japan) was applied to study the crystal structure and phase composition, using a Cu Kα radiation (40 kV, 40 mA) at a glancing angle of 1.5°, in the range of 2θ from 5° to 80° and at a scanning rate of 0.02° s^−1^.

Open circuit potential (OCP) and electrochemical impedance spectra (EIS) were carried out using a CIMPS-2 Zahner system in a three-electrode cell, i.e., a saturated Ag/AgCl reference electrode, a platinum counter electrode and the coated Mg substrate as the working electrode with an exposed area of 1 cm^2^. Before EIS measurements, OCP measurements were performed firstly, and EIS measurements started only when the OCP values kept stable. In addition, the EIS measurements were performed in 3.5 wt% NaCl solution after different immersion times with a sine signal with an amplitude of 10 mV RMS v.s. OCP. The range of measured frequencies extended from 10 MHz to 100 kHz, with a logarithmic sweep of 7 points per decade. The details of characterization steps can be found elsewhere^27,28,30,47^. In order to ensure the reproducibility, there were three parallel samples in each system.

## Results and discussion

**Figure 2:**
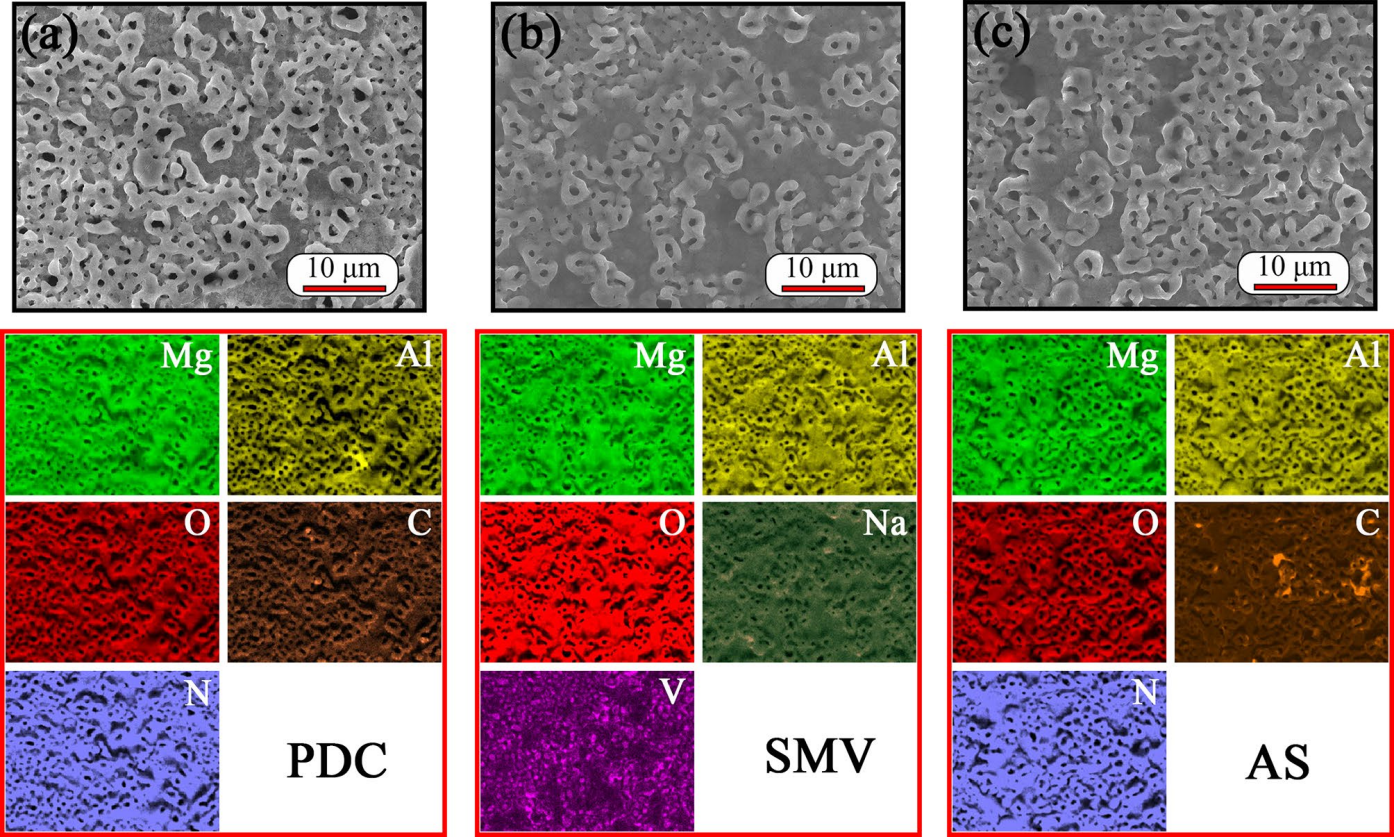
SEM surface morphologies and corresponding EDS mappings of (**a**) PDC, (**b**) SMV and (**c**) AS specimens.

**Figure 3:**
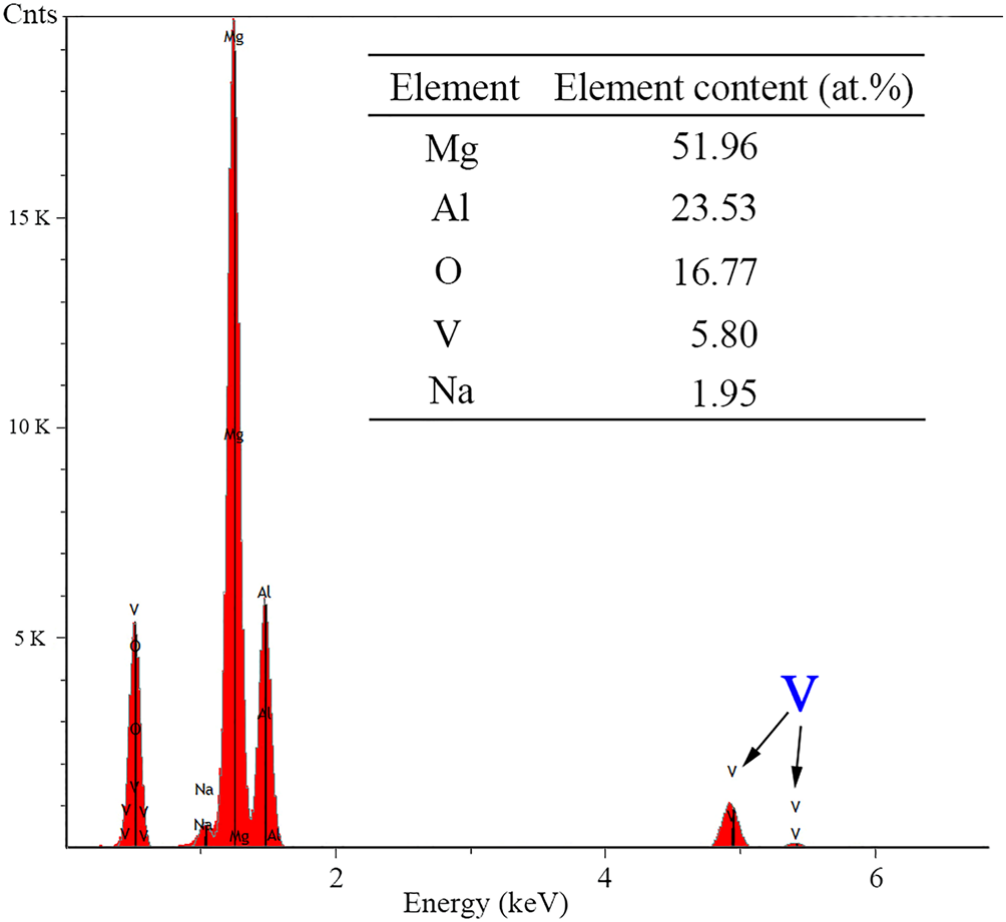
The spectra of point EDS analysis for SMV specimen.

Figure 2 shows SEM surface morphologies and corresponding EDS mappings of PDC, SMV and AS specimens. After impregnation of inhibitors, the surface morphologies for all of specimens did not significantly change, and they still revealed a characteristic surface morphology of the PEO layer, composed by pores with different size and little micro-cracks^7,30^. The formation of such a typical morphology was caused by the gas evolution through the molten oxide during the PEO process and the thermal stresses at the sites of the discharge channel^43^. Usually, electrolyte composition^51^, temperature of electrolyte^52^, concentration of electrolyte^53^, applied energy (voltage and current density)^54,55^, additives (such as TiO_2_, CeO_2_)^56,57^ and secondary phases in the alloy^43^ play important roles in morphologies of PEO coatings including dimensions and distribution of pores and micro-cracks, and further resulting in different performance of PEO coatings. Because of the surface contamination with entrapped and adsorbed CO_2_ and N_2_, it was difficult to determine from EDS mapping if PDC or AS inhibitors had been impregnated successfully into pores of PEO coatings. However, it can be seen clearly from SMV specimens that, there were light spots, which means high element content, in EDS mapping of V. Figure 3 reveals the spectra of point EDS analysis for SMV specimen. From the inserted table in Fig. 3, it shows that the content of V was up to 5.80 at%. All of above results indicate that inhibitors could be successfully impregnated into porous PEO coatings through a vacuuming procedure.

**Figure 4:**
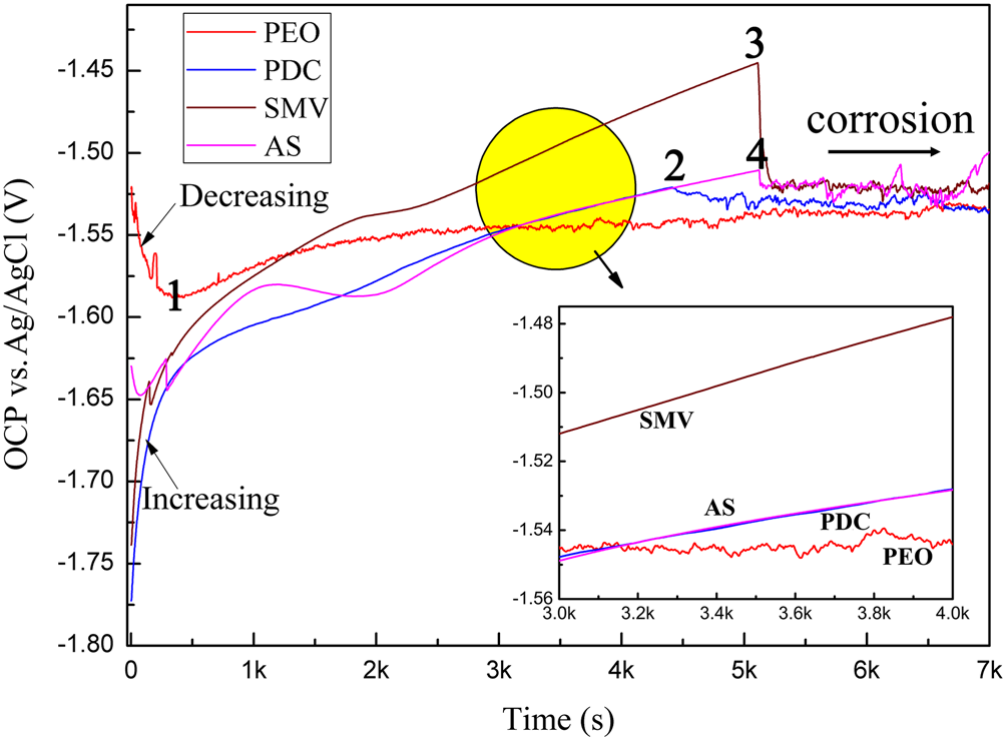
Variation of OCP with immersion time in 3.5 wt% NaCl solution after impregnation of inhibitors.

OCP vs. time measurements in 3.5 wt% NaCl solution after impregnation of inhibitors are shown in Fig. 4. The OCP of PEO specimen was found that the value decreased at the beginning of immersion, which may result from deterioration of PEO coating in such a strong aggressive medium. Subsequently, the OCP value increased gradually (from point 1), due to deposition of corrosion production, likely Mg(OH)_2_. However, it showed a small fluctuation in the OCP value during whole immersion process, which was significantly different from others. Although the OCP values for all of impregnated inhibitor specimens were more negative than PEO specimen in the beginning, continuous and fast potential displacements towards positive values were observed and eventually higher than stable OCP of PEO specimen after a longer immersion term (shown in the insert of Fig. 4). When the immersion time was further prolonged, the OCPs dropped suddenly from point 2, point 3 and point 4 respectively (marked in Fig. 4) and became fluctuating, similar to that of PEO specimen. These results indicated the inhibiting effect of PDC, SMV and AS inhibitors for Mg alloys, while it was very limited when impregnated inhibitor specimens were not sealed.

As for inorganic inhibitor SMV have been proved that it could reduce the kinetics of oxygen reduction to an extent similar to chromates, and a vanadium-based adsorbed layer may form to block reactive sites on intermetallic particles and discourage the oxygen reduction reaction^47,58^. Actually, as for Mg alloy, water reduction is not the only cathodic process and oxygen reduction is the albeit secondary cathodic process during Mg corrosion. Unusually, as for organic inhibitors, either their ligand anions could form stable complexes with cations (e.g. Mg^2+^ in our case) or they could adsorbed physically (electrostatic interaction) between carbonyl functional groups and cations (e.g. Mg^2+^) on metallic surface^59,60^. In Lamaka’s study^15^, as for PDC and AS, it showed almost the same inhibiting efficiency (eg. 83 ± 11% vs. 99 ± 1% for PDC, 86% vs. 85% for AS) for the higher Fe content (CP-Mg-220 ppm) and the lower Fe content (HP-Mg-50 ppm) purity Mg alloys. This result implied that PDC and AS inhibitors mainly interacted with Fe to suppress Mg corrosion. As suggested by their theory^14,61^, at initial cathodic reactions on Fe-rich particles, detachment of Fe particles from the Mg metallic substrate due to anodic dissolution of Mg. Self-corrosion will lead to formation of Fe^3+^/Fe^2+^, and then it reduces and further deposits a thin metallic Fe film, resulting in the increasing in the total cathodic area. Such a replating Fe film at the corrosion forefront drives the cathodic current and becomes the most active H_2_ generator. Hence, some of the iron complexing agents can prevent Fe from Fe replating, and thus compress Mg cathodic reactions. However, not all of the iron complexing agents are effective, at least, it has to be satisfied that their ligands cannot form highly stable soluble complexes with Mg. If not they will accelerate dissolution of Mg although prevention iron re-plaiting. For examples, tartaric acid inhibitor can form highly stable complexes with Fe^3+^ (${log}_{{Fe}^{3+}}^{st}$ = 11.87), most important, the stability of it’s complex with Mg^2+^ is relatively poor (${log}_{{Mg}^{2+}}^{st}$ = 1.91)^62,63^. Actually, although we briefly discuss the inhibiting mechanism of PDC and AS organic inhibitors, it is more complicated and not yet fully clear so far.

**Figure 5:**
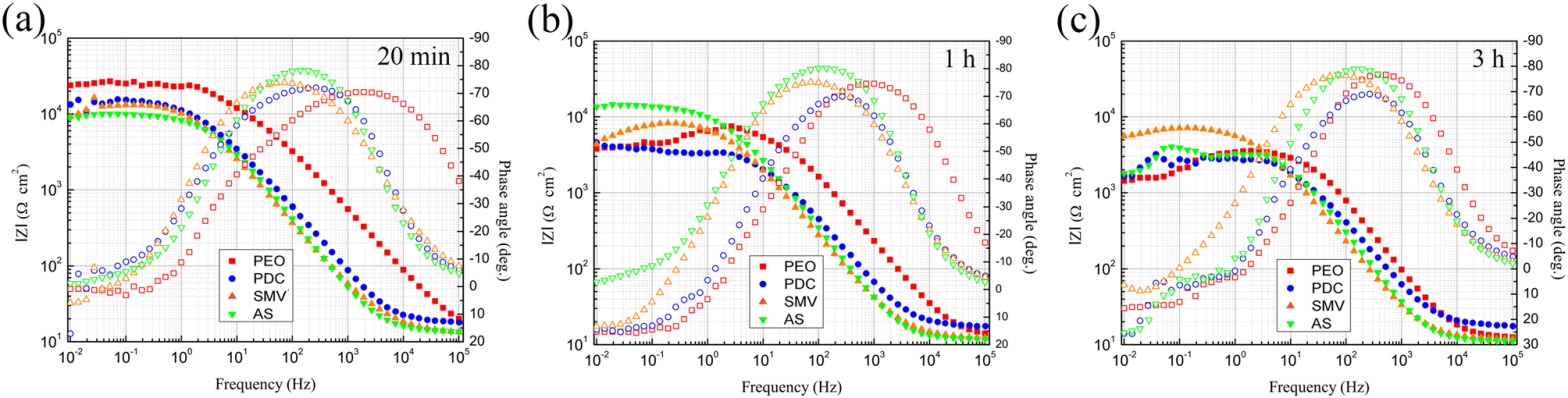
Bode EIS plots of PDC, SMV and AS specimens after (**a**) 20 min, (**b**) 1 h and (**c**) 3 h immersion in 3.5 wt% NaCl solution.

Bode EIS plots of PDC, SMV and AS specimens, shown in Fig. 5, were recorded after 20 min, 1 h and 3 h immersion in 3.5 wt% NaCl solution. It can be seen that two well defined time constants exist in the frequency-phase angle diagram, corresponding PEO coatings and corrosion process. In general, the low-frequency impedance modulus (*|Z|*_*0.01 Hz*_) reveals the overall resistance of the protective coating^64,65^. Initially, the corrosion protection of PEO specimen was superior to that of impregnated inhibitor specimens. Nevertheless, its protection performance dropped faster with immersion time, and eventually became the worst in corrosion protection (*|Z|*_*0.01 Hz*_ = 1.4 × 10^3^ Ω cm^2^). SMV was found to be most efficient (*|Z|*_*0.01 Hz*_ = 5.6 × 10^3^ Ω cm^2^), in agreement with OCP vs. time result where final OCP value of SMV was noble with respect to the other two. According the EIS results, in summary, impregnated inhibitor did not obviously improve corrosion resistance of PEO specimen, which may attribute to easy loss of inhibitors from pores without sealing post-treatment.

**Figure 6:**
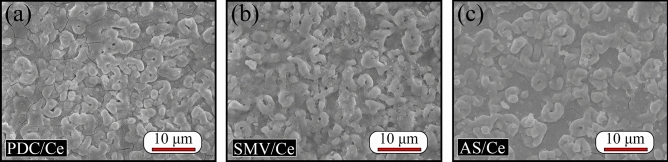
SEM surface morphologies of (**a**) PDC/Ce, (**b**) SMV/Ce and (**c**) AS/Ce specimen.

After sealing treatment in containing Ce solution with short time, SEM surface morphologies of PDC/Ce, SMV/Ce and AS/Ce specimens are presented in Fig. 6. Although the initial morphological characteristics of PEO specimen were still visible (recall Fig. 2), the initial pores of PEO layer were sealed well. But micro-cracks were clearly observed, especially in Fig. 6a, which was obviously not due to PEO treatment and they were new formation during sealing treatment process. Hu et al.^66^ suggested that the micro-cracks only appeared at the thick film, not at the thin film, which is not consistent with our results. This contradictory result seems to be because high temperature and short reaction time was employed in our work for avoiding loss of impregnated inhibitors. In our previous study, We have proposed growth mechanism of cerium conversion coating on PEO layer, i.e., the Ce solution could penetrate into the pores of the PEO layer to precipitate of CeO_2_ or/and Ce(OH)_3_ at the beginning of the treatment, followed by the pores were covered with spherical deposits, and as a result a cracked ‘‘dry mud-like” surface morphology formed^30^. As suggestion, the faster deposition rate caused large stress existed at the PEO coatings, and stress relieve resulted in formation of micro-cracks^67^. A generally accepted opinion, Ce salts precipitated on Mg surface typically showing positive protective performance for Mg alloys^30^. Table 1 lists elemental compositions of PDC/Ce, SMV/Ce and AS/Ce specimens, acquired by the average calculation of the composition of five points on their surface. Higher content of O for PDC/Ce and AS/Ce specimens compared with SMV/Ce specimens and the presence of V for SMV/Ce specimens all indicated that impregnated inhibitors were stored and sealed successfully in porous PEO coatings.

**Table 1:** Elemental compositions of PDC/Ce, SMV/Ce and AS/Ce specimens in Fig. 6.

Element	PDC/Ce	SMV/Ce	AS/Ce
Mg	18.4 ± 4.2	47.5 ± 3.1	20.1 ± 1.2
Al	12.0 ± 2.0	17.5 ± 2.7	13.2 ± 1.4
O	51.5 ± 4.8	25.4 ± 1.5	52.5 ± 0.5
Ce	9.4 ± 2.5	5.5 ± 1.2	6.0 ± 0.6
C	7.0 ± 2.3	–	6.8 ± 0.3
N	1.7 ± 0.5	–	1.4 ± 0.5
Na	–	0.9 ± 0.3	–
V	–	3.2 ± 2.1	–

**Figure 7:**
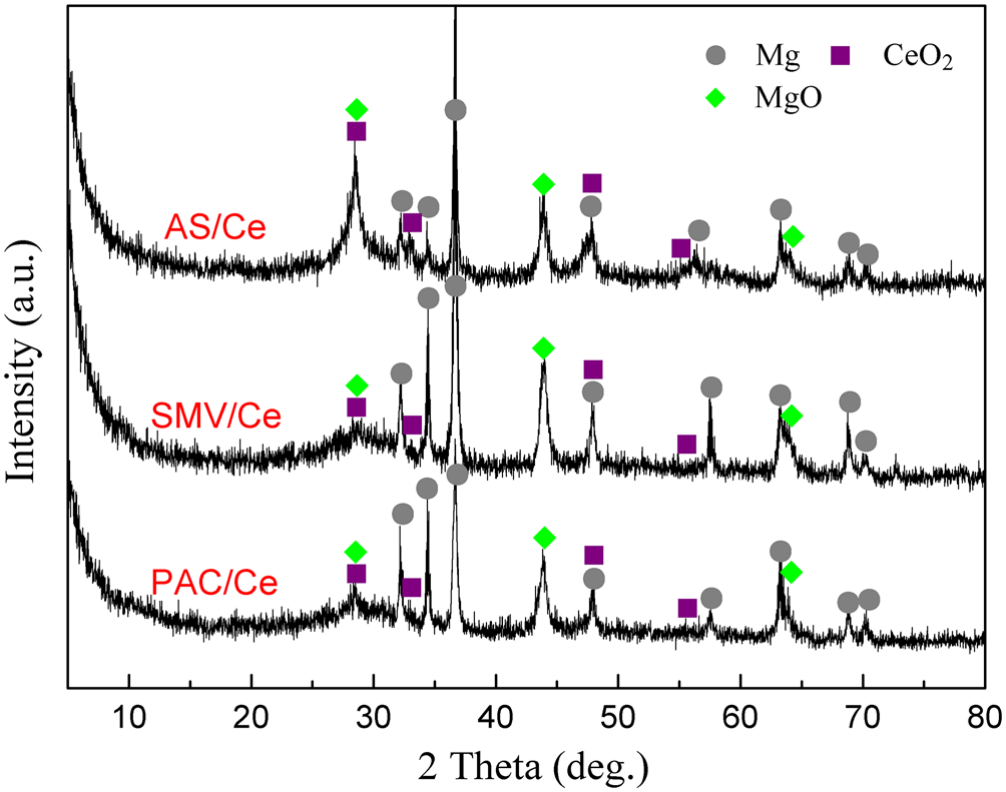
XRD patterns of PDC/Ce, SMV/Ce and AS/Ce specimens.

Figure 7 shows XRD patterns of PDC/Ce, SMV/Ce and AS/Ce specimens. It was found that Ce mainly exists in the form of CeO_2_. Researchers also detected Ce in the 3-valence state (Ce(OH)_3_) after treatment in a Ce containing solution^67,68^. There was no obvious diffraction peaks corresponding to Ce(OH)_3_, attributed to the fact that the the Ce(III) hydroxide may present in an amorphous or nano-crystalline form. The transform and formation process can be expressed by following reactions^30^:

1 $$ {\text{H}}_{{2}} {\text{O}}_{{2}} \left( {{\text{aq}}} \right) + {\text{2e}}^{ - } \to {\text{2OH}}^{ - } \;\left( {{\text{aq}}} \right) $$

2 $$ {\text{2Ce}}^{{{3} + }} \;\left( {{\text{aq}}} \right) + {\text{H}}_{{2}} {\text{O}}_{{2}} \;\left( {{\text{aq}}} \right) + {\text{2OH}}^{ - } \;\left( {{\text{aq}}} \right) \to {\text{2Ce}}\;\left( {{\text{OH}}} \right)_{2}^{2 + } \;\left( {{\text{aq}}} \right) $$

3 $$ {\text{2Ce}}\;\left( {{\text{OH}}} \right)_{2}^{2 + } \left( {{\text{aq}}} \right) + {\text{2OH}}^{ - } \left( {{\text{aq}}} \right) \to {\text{CeO}}_{{2}} \cdot {\text{H}}_{{2}} {\text{O}}\left( {\text{s}} \right) + {\text{H}}_{{2}} {\text{O}} $$

4 $$ {\text{2Ce}}\left( {{\text{OH}}} \right)_{2}^{2 + } \;\left( {{\text{aq}}} \right) + {\text{2 OH}}^{ - } \;\left( {{\text{aq}}} \right) \to {\text{CeO}}_{{2}} \;\left( {\text{s}} \right) + {\text{2H}}_{{2}} {\text{O}} $$

**Figure 8:**
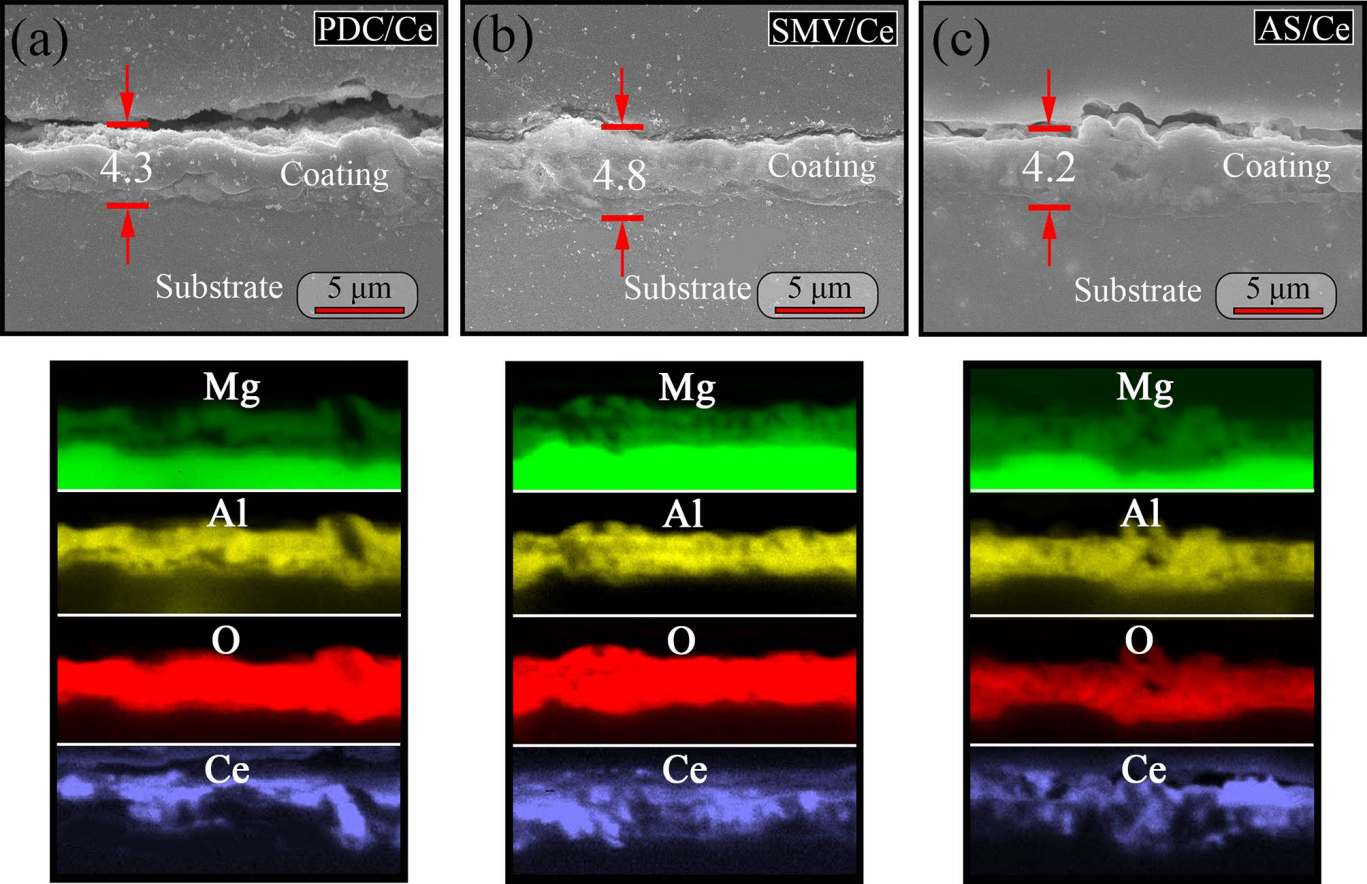
The cross-sectional SEM images and corresponding EDS mappings of (**a**) PDC/Ce, (**b**) SMV/Ce and (**c**) AS/Ce specimens.

Figure 8 shows the cross-sectional SEM images and corresponding EDS mappings of PDC/Ce, SMV/Ce and AS/Ce specimens. According our data, the thickness of PEO layer was about 5.0 ± 0.4 μm (not shown here). Thus it can be seen that treatment in containing Ce solution did not significantly change the thickness of PEO coatings (4.0 ± 0.6 μm for PDC/Ce, 4.5 ± 1.0 μm for SMV/Ce and 4.2 ± 0.8 μm for AS/Ce). Furthermore, it was found that all of coatings were relative thin and non-uniform, which is not beneficial to improve of corrosion resistance. From EDS mappings, Mg, Al and O were present throughout the oxide thickness down to the interface between substrate and oxide, while the position of rich Ce closed to outer layer. So far, the inhibiting effect of Ce toward Mg alloys is still in debate. As suggested by Lamaka et al.^15^, CeCl_3_ would accelerate corrosion for most alloys, e.g. WE43, ZE41, E21, AZ31, AZ91 and AM50, while a completely opposite result obtained from Ce(NO_3_)_3_ on AZ63, AM50 and AM60^15,69,70^. Hence, some of them proposed that the inhibiting effects of Ce(NO_3_)_3_ were resorted to the presence of nitrate, rather than Ce ions. However, in our published work, it has been proved that presence of Ce ion in coatings was believed to endow the coating with active corrosion protection, but its protective effect was still limited^30^.

**Figure 9:**
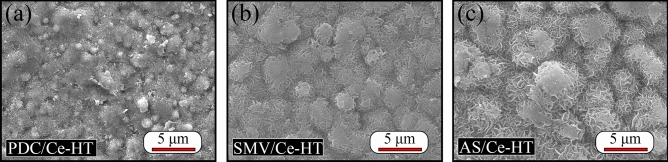
The SEM surface morphologies of (**a**) PDC/Ce-HT, (**b**) SMV/Ce-HT and (**c**) AS/Ce-HT specimens.

**Figure 10:**
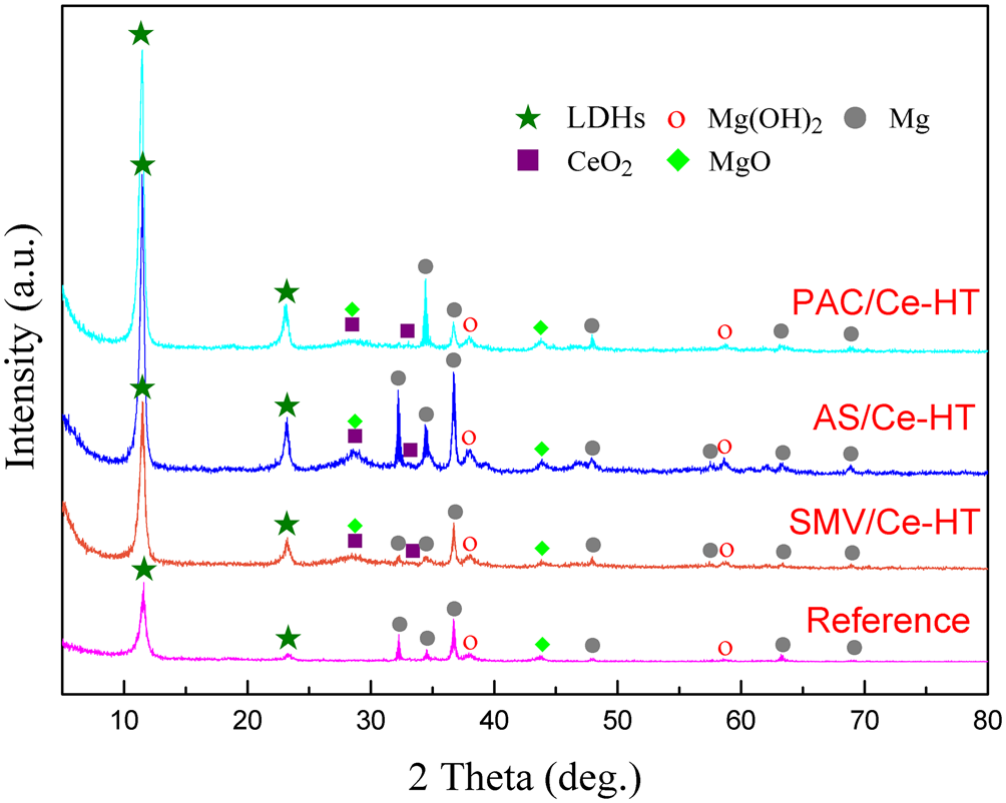
XRD patterns of PDC/Ce-HT, SMV/Ce-HT, AS/Ce-HT and reference specimens.

After fabrication of MgAl-LDHs on PDC/Ce, SMV/Ce and AS/Ce specimens, the SEM surface morphologies are revealed in Fig. 9. The original PEO layer completely fragmented, resulting in the formation of island-like structure covered by tiny nanoflakes, and thus original morphology of PEO layer was still faintly visible. These tiny nanoflakes was generally considered to be the characteristic structures of LDHs in many research cases^36–41^. It is worth mention that Mg(OH)_2_ reveals a layered structure as well, attributed that MgAl-LDHs unusually originates from the transformation of Mg(OH)_2_ or Al(OH)_3_^48^. In the strict sense, it's not accurate to confirm presence of LDHs through only SEM image. Thus, XRD measurement was further carried out and the result was shown in Fig. 10. In order to clarify if impregnation of inhibitors has an influence (positive or negative) on subsequent LDHs growth, a reference specimen was produced via PEO specimen with Ce solution sealing treatment, followed by preparation of LDHs on them (without impregnation of inhibitors into porous PEO coatings) and the XRD result of this reference specimen is also shown in Fig. 10. The diffraction peaks associated with impurity phase Mg(OH)_2_ were visible. In addition to Mg(OH)_2_, all of specimens appeared the characteristic diffraction peaks of LDHs and the characteristic 003 and 006 planes located at about 11.3° and 22.2°, indicating that their interlayers are loaded with hydroxides. In contrast, all of the characteristic diffraction of LDHs peaks for PDC/Ce-HT, SMV/Ce-HT and AS/Ce-HT were stronger than that of the reference specimen. That means that impregnation of inhibitors into porous PEO coatings has played a positive role in improving LDHs content of final composite coatings. Recently, Shulha et al.^71^ studied organic chelating agent-assisted in situ LDH growth on AZ31 Mg alloy. As suggested by their theory, the pH range mostly suitable for MgAl-LDH formation was between 9 and 10, while an excess of OH^-^ developed in the solution when pH increased to above 9.5, resulting in the formation of Mg(OH)_2_. After addition of chelating agent EDTA, the concentration of Mg-EDTA complex (MgC_10_H_12_N_2_O_8_^2−^) reached its maximum at a pH from 7 to 10.5. Thus, the introduction of EDTA delayed the formation of Mg(OH)_2_ by 0.8 pH units, i.e., only when the pH exceeds 10.3 could form Mg(OH)_2_ (9.5 for that without chelating agent). Similar to EDTA, ligand anions would compete with OH^-^ ions at a pH from 4.5 to 13.8 when chelating agent NTA was introduced, resulting in formation of Mg-NTA complex (Mg(CH_6_O_6_N)_2_^4−^). In a word, they proposed that formation of MgAl-LDH could be promoted via the maintenance of free Mg^2+^ or Mg reversibly bound in Mg-ligand complexes at pH exceeding typical precipitation range of Mg(OH)_2_. Back to our work, it has been previously shown that ligand anions of PDC and AS could form stable complexes with metal cations (e.g. Mg^2+^ and Fe^3+^)^62,63^. Therefore, there was reason to believe that a similar effect has taken place in PDC and AS, which also would further promote the formation of LDHs. And the lowest peak intensity of inorganic inhibitor SMV, compared with PDC and AS, also indirectly prove our opinion. However, further experiments are necessary to verify our guess.

**Figure 11:**
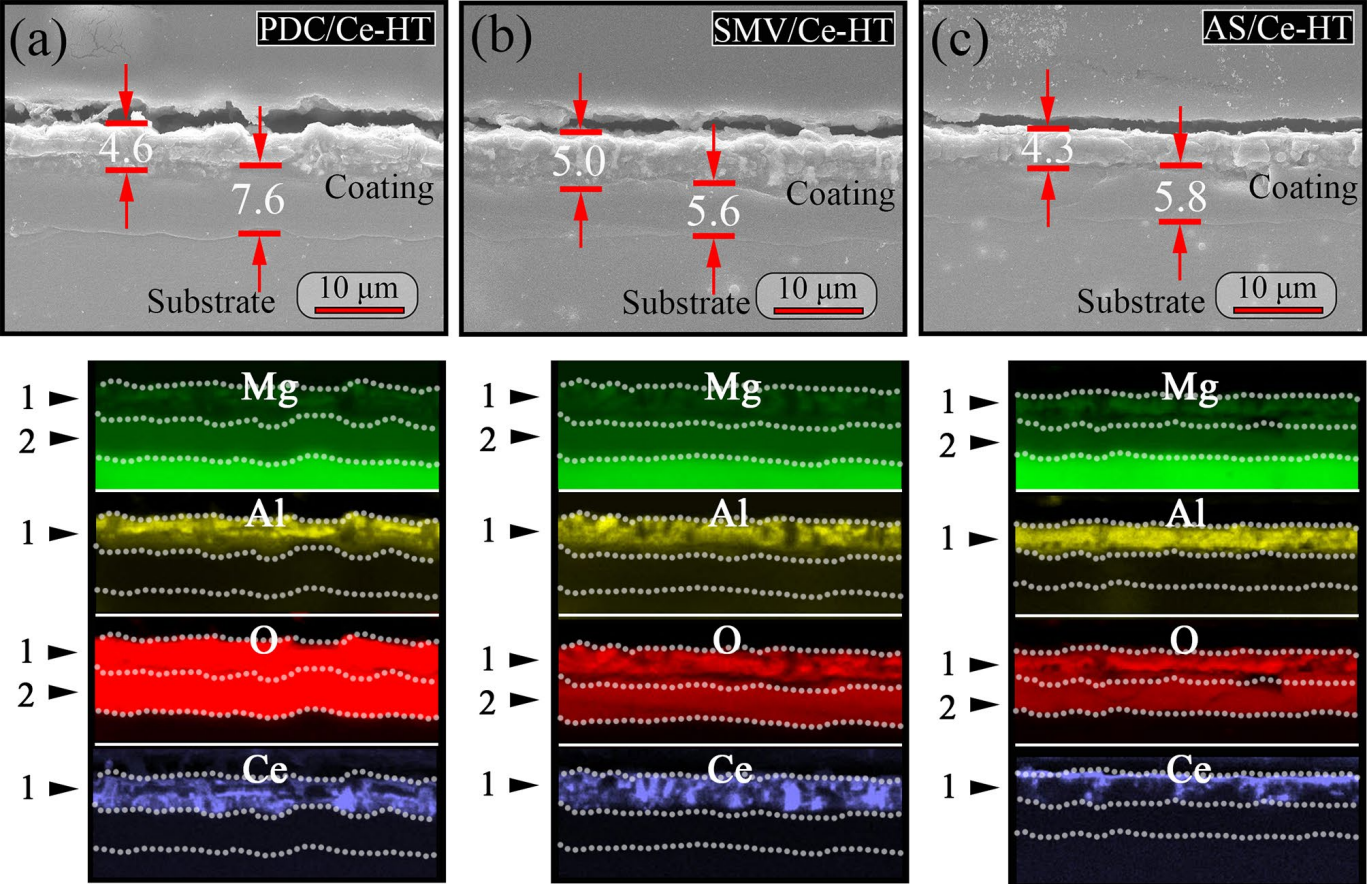
The cross-sectional SEM images and corresponding EDS mappings of (**a**) PDC/Ce-HT, (**b**) SMV/Ce-HT and (**c**) AS/Ce-HT specimens.

**Figure 12:**
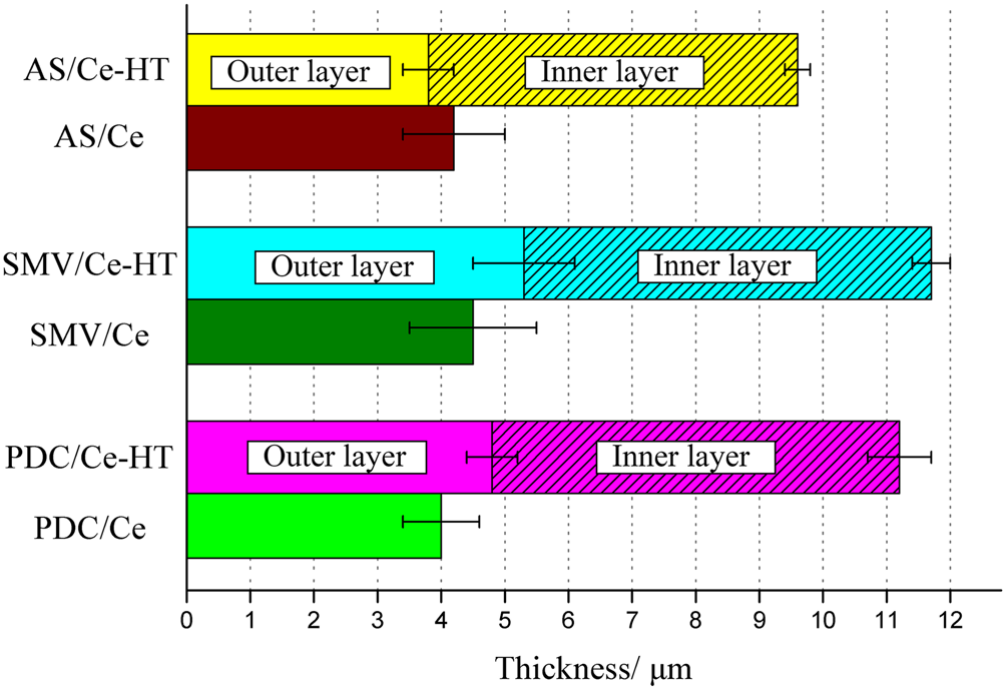
Evolution of total coating thickness.

Figure 11 shows the cross-sectional SEM images and corresponding EDS mappings of PDC/Ce-HT, SMV/Ce-HT and AS/Ce-HT specimens. Different from Fig. 8, the results of EDS mapping clearly revealed that all composite coatings were consist of typically two-layers structure, which is consistent with our previous result^65^. The evolution of average thickness of the coating obtained by the cross-sectional SEM micrograph is plotted in Fig. 12. It is clearly seen, that the thickness of outer layer of PDC/Ce-HT, SMV/Ce-HT and AS/Ce-HT specimens was almost the same as respective thickness of original specimens without preparation of LDHs. This result implied that severe damage of PEO layer did not take place during twice hydrothermal process. In addition, the thickness of coatings significantly increased after fabrication of LDHs, with the thickest one being 11.7 μm (SMV/Ce-HT).

**Figure 13:**
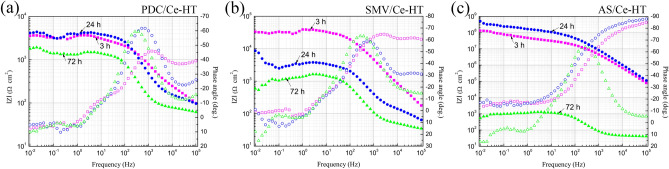
Bode EIS plots of (**a**) PDC/Ce-HT, (**b**) SMV/Ce-HT and (**c**) AS/Ce-HT specimens after 3 h, 24 h and 72 h immersion in 3.5 wt% NaCl solution.

**Figure 14:**
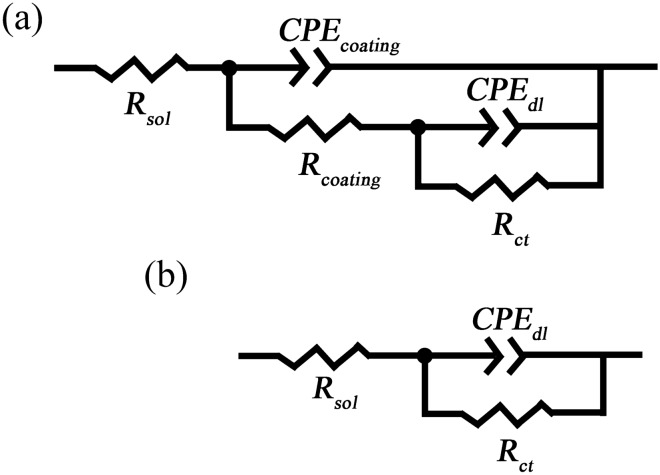
Equivalent circuits used to fit the EIS spectra.

Figure 13 presents Bode EIS plots of PDC/Ce-HT, SMV/Ce-HT and AS/Ce-HT specimens, recorded after 3 h, 24 h and 72 h immersion in 3.5 wt% NaCl solution. According to the low-frequency impedance modulus (*|Z|*_*0.01 Hz*_) at initial immersion stage (3 h), the corrosion resistance can be ranked in increasing order as follows: PDC/Ce-HT (*|Z|*_*0.01 Hz*_ = 3.5 × 10^3^ Ω cm^2^) < SMV/Ce-HT (*|Z|*_*0.01 Hz*_ = 3.3 × 10^4^ Ω cm^2^) ≪ AS/Ce-HT (*|Z|*_*0.01 Hz*_ = 1.3 × 10^8^ Ω cm^2^). From frequency-impedance modulus diagram, as for organic inhibitor PDC and AS, the impedance modulus in both higher frequency and lower frequency slightly increased (from 3 h immersion to 6 h immersion) and reached a maximum after ca. 6 h immersion, which may be correlated with impregnated inhibitors resulting in formation of renewed production, resulting in a sealing effect to corrosion defects and active corrosion of LDHs. In contrast, the impedance modulus of SMV/Ce-HT specimens dropped faster from 3 to 6 h immersion. The immersion time was further prolonged to 72 h, all of specimens decrease in the impedance modulus. In summary, in terms of overall corrosion resistance, AS/Ce-HT specimen was best. As a result, equivalent circuits, shown in Fig. 14, were employed to further fit the electrical parameters of AS/Ce-HT specimen. Due to the nonhomogeneity, caused probably by heterogeneity of chemical composition, the degree of crystallinity and stoichiometry, and the presence of the space-charge region, the constant phase elements (CPEs) were used herein to demonstrate the non-ideal resistive and capacitive behavior of the coated specimens^72,73^. In the equivalent circuit, *R*_*sol*_ corresponds to the solution resistance; *R*_*coating*_ and *CPE*_*coating*_ are the resistive and capacitive response of the coating. When the aggressive media (such as Cl^-^ in our case) diffuses through coating and reaches to the interface between coating and substrate, Mg substrate will be corroded. The corrosion process of Mg substrate can be characterized by the double layer capacitance of the electrolyte/substrate interface (*CPE*_*dl*_) and charge transfer resistance (*R*_*ct*_)^30,74–77^.

**Figure 15:**
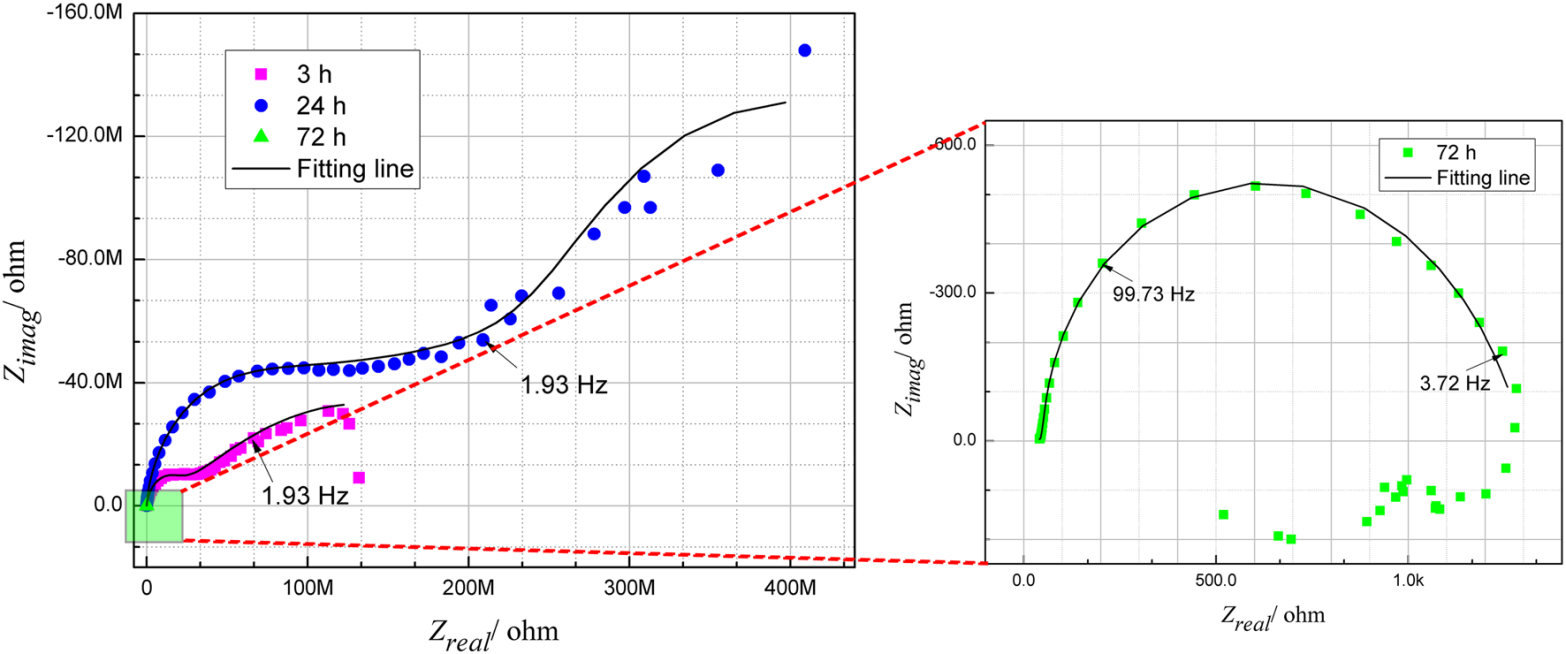
Nyquist plot of AS/Ce-HT specimen and the fitted result using equivalent circuits shown in Fig. 14.

Figure 15 shows nyquist plot of AS/Ce-HT specimen. At initial and medium immersion stage (3 h and 24 h), two capacitive arcs corresponding to two well-defined time constants were observed. Therefore, the equivalent circuit shown in Fig. 14a was used to fit resistive and capacitive of the coating and corrosion process. After immersion of 72 h, there were only one capacitive arc left and its radius dropped fast, indicating the coating barrier effect completely lost at this time. Therefore, only one resistance and capacitance in parallel circuit (Fig. 14b) was used to fit resistive and capacitive of corrosion process. The fitted result is revealed in Fig. 15 as well and the chi-square *χ*^*2*^ for all fitting lines was reached less than 10^–2^. Table 2 lists the fitting electrical parameters of AS/Ce-HT specimen. Compared with our previous data, this kind of coating own a higher *R*_*ct*_ value at initial immersion stage under same test condition (most of them were in between 10^4^ and 10^6^), indicating a superior barrier effect against corrosion^27,47,65,78^.

**Table 2:** Fitted parameters for EIS spectra of AS/Ce-HT specimen depicted in Fig. 15.

Immersion time	*C*_*coating*_ (S s^n^ cm^-2^)	*n*_*coating*_	*R*_*coating*_ (Ω cm^2^)	*C*_*dl*_ (S s^n^ cm^-2^)	*n*_*dl*_	*R*_*ct*_ (Ω cm^2^)	*χ*^2^
3 h	9.1 × 10^–11^	0.8	1.6 × 10^7^	1.3 × 10^–8^	0.3	2.4 × 10^8^	5.5 × 10^–3^
24 h	2.8 × 10^–11^	0.9	4.1 × 10^8^	4.2 × 10^–9^	0.2	1.6 × 10^12^	5.3 × 10^–3^
72 h	–	–	–	4.5 × 10^–6^	0.9	1.2 × 10^3^	3.7 × 10^–3^

## Conclusion

1. Corrosion inhibitors PDC, SMV and AS were successfully impregnated into the micro pores of PEO layer through a vacuuming procedure, however, impregnated inhibitor did not obviously improve their corrosion resistance.
2. After sealing treatment with short time in Ce containing solution, the formation of new phase, CeO_2_, sealed inhibitors stored pores of PEO, while a small amount of inhibitor also lost in this process.
3. When LDHs was prepared on PEO layer sealed by Ce conversion coating, resulting in formation of island-like structure covered by tiny nanoflakes of LDHs. And, the structure of the coatings varied from one layer to two layers and the thickness significantly improved, with the thickest one being 11.7 μm (SMV/Ce-HT). Moreover, it was found that impregnation of inhibitors into porous PEO coatings has played a positive role in improving LDHs content of final coatings.
4. Eventually, EIS result indicated that AS/Ce-HT exhibited a superior corrosion protection, better than other coatings. In addition, further loading inhibitors into LDHs can be considered as a next step.

## References

[1] Arthanari S, Nallaiyan R, Kwang Seon S (2017). Electrochemical corrosion behavior of acid treated strip cast AM50 and AZX310 magnesium alloys in 3.5 w.t% NaCl solution. J. Magnes. Alloys.

[2] Song GL (2013). Corrosion Behavior and Prevention Strategies for Magnesium (Mg) Alloys.

[3] Adsul SH, Soma Raju KRC, Sarada BV, Sonawane SH, Subasri R (2018). Evaluation of self-healing properties of inhibitor loaded nanoclay-based anticorrosive coatings on magnesium alloy AZ91D. J. Magnes. Alloys.

[4] Du W, Liu K, Ma K, Wang Z, Li S (2018). Effects of trace Ca/Sn addition on corrosion behaviors of biodegradable Mg–4Zn–0.2Mn alloy. J. Magnesium Alloys.

[5] Atrens A, Song G-L, Liu M, Shi Z, Cao F, Dargusch MS (2015). Review of Recent Developments in the Field of Magnesium Corrosion. Adv. Eng. Mater..

[6] Blawert C, Dietzel W, Ghali E, Song G (2006). Anodizing treatments for magnesium alloys and their effect on corrosion resistance in various environments. Adv. Eng. Mater..

[7] Farhadi SS, Aliofkhazraei M, Barati Darband G, Abolhasani A, Sabour Rouhaghdam A (2017). Corrosion and wettability of PEO coatings on magnesium by addition of potassium stearate. J. Magnes. Alloys.

[8] Lu X, Blawert C, Huang Y, Ovri H, Zheludkevich ML, Kainer KU (2016). Plasma electrolytic oxidation coatings on Mg alloy with addition of SiO2 particles. Electrochim. Acta.

[9] Dias SAS, Lamaka SV, Nogueira CA, Diamantino TC, Ferreira MGS (2012). Sol–gel coatings modified with zeolite fillers for active corrosion protection of AA2024. Corros. Sci..

[10] Zhang G, Wu L, Tang A, Ding X, Jiang B, Atrens A, Pan F (2019). Smart epoxy coating containing zeolites loaded with Ce on a plasma electrolytic oxidation coating on Mg alloy AZ31 for active corrosion protection. Prog. Org. Coat..

[11] Chen XB, Birbilis N, Abbott TB (2011). Review of corrosion-resistant conversion coatings for magnesium and its alloys. Corrosion.

[12] Mei D, Lamaka SV, Gonzalez J, Feyerabend F, Willumeit-Römer R, Zheludkevich ML (2019). The role of individual components of simulated body fluid on the corrosion behavior of commercially pure Mg. Corros. Sci..

[13] Frignani A, Grassi V, Zanotto F, Zucchi F (2012). Inhibition of AZ31 Mg alloy corrosion by anionic surfactants. Corros. Sci..

[14] Lamaka SV, Höche D, Petrauskas RP, Blawert C, Zheludkevich ML (2016). A new concept for corrosion inhibition of magnesium: Suppression of iron re-deposition. Electrochem. Commun..

[15] Lamaka SV, Vaghefinazari B, Mei D, Petrauskas RP, Höche D, Zheludkevich ML (2017). Comprehensive screening of Mg corrosion inhibitors. Corros. Sci..

[16] Han Z, Chen H, Zhou S (2017). Dissociation and diffusion of hydrogen on defect-free and vacancy defective Mg (0001) surfaces: A density functional theory study. Appl. Surf. Sci..

[17] Hughes AE, Mol JMC, Zheludkevich ML, Buchheit RG (2016). Active Proctive Coatings.

[18] Gnedenkov AS, Sinebryukhov SL, Mashtalyar DV, Gnedenkov SV (2016). Localized corrosion of the Mg alloys with inhibitor-containing coatings: SVET and SIET studies. Corros. Sci..

[19] Sun M, Yerokhin A, Bychkova MY, Shtansky DV, Levashov EA, Matthews A (2016). Self-healing plasma electrolytic oxidation coatings doped with benzotriazole loaded halloysite nanotubes on AM50 magnesium alloy. Corros. Sci..

[20] Gnedenkov AS, Sinebryukhov SL, Mashtalyar DV, Gnedenkov SV (2016). Protective properties of inhibitor-containing composite coatings on a Mg alloy. Corros. Sci..

[21] Zhang F, Ju P, Pan M, Zhang D, Huang Y, Li G, Li X (2018). Self-healing mechanisms in smart protective coatings: A review. Corros. Sci..

[22] White SR (2001). Autonomic healing of polymer composites. Lett. Nat..

[23] Maia F, Tedim J, Lisenkov AD, Salak AN, Zheludkevich ML, Ferreira MGS (2012). Silica nanocontainers for active corrosion protection. Nanoscale.

[24] Shchukin DG, Zheludkevich M, Yasakau K, Lamaka S, Ferreira MGS, Möhwald H (2006). Layer-by-Layer Assembled Nanocontainers for Self-Healing Corrosion Protection. Adv. Mater..

[25] Kartsonakis I, Daniilidis I, Kordas G (2008). Encapsulation of the corrosion inhibitor 8-hydroxyquinoline into ceria nanocontainers. J. Sol-Gel Sci. Technol..

[26] Liu X, Gu C, Wen Z, Hou B (2018). Improvement of active corrosion protection of carbon steel by water-based epoxy coating with smart CeO_2_ nanocontainers. Prog. Org. Coat..

[27] Zhang G, Wu L, Tang A, Weng B, Atrens A, Ma S, Liu L, Pan F (2018). Sealing of anodized magnesium alloy AZ31 with MgAl layered double hydroxides layers. RSC Adv..

[28] Wu L, Yang D, Zhang G, Zhang Z, Zhang S, Tang A, Pan F (2018). Fabrication and characterization of Mg-M layered double hydroxide films on anodized magnesium alloy AZ31. Appl. Surf. Sci..

[29] Abdolah Zadeh M, Tedim J, Zheludkevich M, van der Zwaag S, Garcia SJ (2018). Synergetic active corrosion protection of AA2024-T3 by 2D- anionic and 3D-cationic nanocontainers loaded with Ce and mercaptobenzothiazole. Corros. Sci..

[30] Zhang G, Wu L, Tang A, Ma Y, Song G-L, Zheng D, Jiang B, Atrens A, Pan F (2018). Active corrosion protection by a smart coating based on a MgAl-layered double hydroxide on a cerium-modified plasma electrolytic oxidation coating on Mg alloy AZ31. Corros. Sci..

[31] Yang J, Blawert C, Lamaka SV, Snihirova D, Lu X, Di S, Zheludkevich ML (2018). Corrosion protection properties of inhibitor containing hybrid PEO-epoxy coating on magnesium. Corros. Sci..

[32] Lamaka SV, Knörnschild G, Snihirova DV, Taryba MG, Zheludkevich ML, Ferreira MGS (2009). Complex anticorrosion coating for ZK30 magnesium alloy. Electrochim. Acta.

[33] Pezzato L, Babbolin R, Cerchier P, Marigo M, Dolcet P, Dabalà M, Brunelli K (2020). Sealing of PEO coated AZ91magnesium alloy using solutions containing neodymium. Corros. Sci..

[34] Mingo B, Arrabal R, Mohedano M, Llamazares Y, Matykina E, Yerokhin A, Pardo A (2018). Influence of sealing post-treatments on the corrosion resistance of PEO coated AZ91 magnesium alloy. Appl. Surf. Sci..

[35] Pezzato L, Coelho LB, Bertolini R (2019). Corrosion and mechanical properties of plasma electrolytic oxidation-coated AZ80 magnesium alloy. Mater. Corros..

[36] Li W, Liu A, Tian H, Wang D (2018). Controlled release of nitrate and molybdate intercalated in Zn-Al-layered double hydroxide nanocontainers towards marine anticorrosion applications. Colloid Interface Sci. Commun..

[37] Alibakhshi E, Ghasemi E, Mahdavian M, Ramezanzadeh B (2017). A comparative study on corrosion inhibitive effect of nitrate and phosphate intercalated Zn-Al- layered double hydroxides (LDHs) nanocontainers incorporated into a hybrid silane layer and their effect on cathodic delamination of epoxy topcoat. Corros. Sci..

[38] Dong Q, Ba Z, Jia Y, Chen Y, Lv X, Zhang X, Wang Z (2017). Effect of solution concentration on sealing treatment of Mg-Al hydrotalcite film on AZ91D Mg alloy. J. Magnes. Alloys.

[39] Chen J, Song Y, Shan D, Han E-H (2013). Modifications of the hydrotalcite film on AZ31 Mg alloy by phytic acid: The effects on morphology, composition and corrosion resistance. Corros. Sci..

[40] Alibakhshi E, Ghasemi E, Mahdavian M, Ramezanzadeh B, Farashi S (2016). Fabrication and characterization of PO43−intercalated Zn-Al-layered double hydroxide nanocontainer. J. Electrochem. Soc..

[41] Tedim J, Poznyak SK, Kuznetsova A, Raps D, Hack T, Zheludkevich ML, Ferreira MG (2010). Enhancement of active corrosion protection via combination of inhibitor-loaded nanocontainers. ACS Appl. Mater. Interfaces.

[42] Serdechnova M, Mohedano M, Kuznetsov B, Mendis CL, Starykevich M, Karpushenkov S, Tedim J, Ferreira MGS, Blawert C, Zheludkevich ML (2017). PEO coatings with active protection based on in-situ formed LDH-nanocontainers. J. Electrochem. Soc..

[43] Mohedano M, Serdechnova M, Starykevich M, Karpushenkov S, Bouali AC, Ferreira MGS, Zheludkevich ML (2017). Active protective PEO coatings on AA2024: Role of voltage on in-situ LDH growth. Mater. Des..

[44] Peng F, Wang D, Tian Y, Cao H, Qiao Y, Liu X (2017). Sealing the Pores of PEO Coating with Mg-Al Layered Double Hydroxide: Enhanced Corrosion Resistance, Cytocompatibility and Drug Delivery Ability. Sci. Rep..

[45] Dou B, Wang Y, Zhang T, Liu B, Shao Y, Meng G, Wang F (2016). Growth behaviors of layered double hydroxide on microarc oxidation film and anti-corrosion performances of the composite film. J. Electrochem. Soc..

[46] Serdechnova M, Mohedano M, Bouali A, Höche D, Kuznetsov B, Karpushenkov S, Blawert C, Zheludkevich M (2017). Role of phase composition of PEO coatings on AA2024 for in-situ LDH growth. Coatings.

[47] Zhang G, Wu L, Tang A, Zhang S, Yuan B, Zheng Z, Pan F (2017). A novel approach to fabricate protective layered double hydroxide films on the surface of anodized Mg-Al alloy. Adv. Mater. Interfaces.

[48] Wu L, Zhang G, Tang A, Liu Y, Atrens A, Pan F (2017). Communication: Fabrication of protective layered double hydroxide films by conversion of anodic films on magnesium alloy. J. Electrochem. Soc..

[49] Zhang G, Wu L, Tang A, Chen X-B, Ma Y, Long Y, Peng P, Ding X, Pan H, Pan F (2018). Growth behavior of MgAl-layered double hydroxide films by conversion of anodic films on magnesium alloy AZ31 and their corrosion protection. Appl. Surf. Sci..

[50] Petrova E, Serdechnova M, Shulha T, Lamaka SV, Wieland DCF, Karlova P, Blawert C, Starykevich M, Zheludkevich ML (2020). Use of synergistic mixture of chelating agents for in situ LDH growth on the surface of PEO-treated AZ91. Sci. Rep..

[51] Mori Y, Koshi A, Liao J, Asoh H, Ono S (2014). Characteristics and corrosion resistance of plasma electrolytic oxidation coatings on AZ31B Mg alloy formed in phosphate: Silicate mixture electrolytes. Corros. Sci..

[52] Chai L, Yu X, Yang Z, Wang Y, Okido M (2008). Anodizing of magnesium alloy AZ31 in alkaline solutions with silicate under continuous sparking. Corros. Sci..

[53] Luo H, Cai Q, Wei B, Yu B, Li D, He J, Liu Z (2008). Effect of (NaPO3)6 concentrations on corrosion resistance of plasma electrolytic oxidation coatings formed on AZ91D magnesium alloy. J. Alloys Compd..

[54] Zhu W, Fang Y-J, Zheng H, Tan G, Cheng H, Ning C (2013). Effect of applied voltage on phase components of composite coatings prepared by micro-arc oxidation. Thin Solid Films.

[55] Bala Srinivasan P, Liang J, Blawert C, Störmer M, Dietzel W (2009). Effect of current density on the microstructure and corrosion behaviour of plasma electrolytic oxidation treated AM50 magnesium alloy. Appl. Surf. Sci..

[56] Mohedano M, Blawert C, Zheludkevich ML (2015). Silicate-based plasma electrolytic oxidation (PEO) coatings with incorporated CeO2 particles on AM50 magnesium alloy. Mater. Des..

[57] Lu X, Mohedano M, Blawert C, Matykina E, Arrabal R, Kainer KU, Zheludkevich ML (2016). Plasma electrolytic oxidation coatings with particle additions: A review. Surf. Coat. Technol..

[58] Iannuzzi M, Frankel GS (2007). Mechanisms of corrosion inhibition of AA2024-T3 by vanadates. Corros. Sci..

[59] Gomma GK (1998). Mechanism of corrosion behaviour of carbon steel in tartaric and malic acid in the presence of Fe^2+^ ion. Mater. Chem. Phys..

[60] Qiang Y, Guo L, Zhang S, Li W, Yu S, Tan J (2016). Synergistic effect of tartaric acid with 2,6-diaminopyridine on the corrosion inhibition of mild steel in 0.5 M HCl. Sci. Rep..

[61] Hoche D, Blawert C, Lamaka SV, Scharnagl N, Mendis C, Zheludkevich ML (2016). The effect of iron re-deposition on the corrosion of impurity-containing magnesium. Phys. Chem. Chem. Phys..

[62] Napoli A (1972). Complex formation of iron(III) with diglycolic and iminodiacetic acids. J. Inorg. Nucl. Chem..

[63] Timberlake CF (1964). Iron-tartrate complexes. J. Chem. Soc..

[64] Tedim J, Bastos AC, Kallip S, Zheludkevich ML, Ferreira MGS (2016). Corrosion protection of AA2024-T3 by LDH conversion films. Analysis of SVET results. Electrochim. Acta.

[65] Zhang G, Wu L, Tang A, Pan H, Ma Y, Zhan Q, Tan Q, Pan F, Atrens A (2018). Effect of micro-arc oxidation coatings formed at different voltages on the in situ growth of layered double hydroxides and their corrosion protection. J. Electrochem. Soc..

[66] Hu J, Tang S, Zhang Z (2008). Microstructure and formation mechanism of cerium conversion coating on alumina borate whisker-reinforced AA6061 composite. Corros. Sci..

[67] Valdez B, Kiyota S, Stoytcheva M, Zlatev R, Bastidas JM (2014). Cerium-based conversion coatings to improve the corrosion resistance of aluminium alloy 6061–T6. Corros. Sci..

[68] Scholes FH, Soste C, Hughes AE, Hardin SG, Curtis PR (2006). The role of hydrogen peroxide in the deposition of cerium-based conversion coatings. Appl. Surf. Sci..

[69] Heakal FE, Shehata OS, Tantawy NS (2012). Enhanced corrosion resistance of magnesium alloy AM60 by cerium(III) in chloride solution. Corros. Sci..

[70] Chen L, Chen CG, Wang NN, Wang JM, Deng L (2015). Study of cerium and lanthanum conversion coatings on AZ63 magnesium alloy surface. Rare Metal Mater. Eng..

[71] Shulha TN, Serdechnova M, Lamaka SV, Wieland DCF, Lapko KN, Zheludkevich ML (2018). Chelating agent-assisted in situ LDH growth on the surface of magnesium alloy. Sci. Rep..

[72] Gnedenkov SV, Sinebryukhov SL, Sergienko VI (2006). Electrochemical impedance simulation of a metal oxide heterostructure/electrolyte interface: A review. Russ. J. Electrochem..

[73] Wu L, Ding X, Zheng Z, Ma Y, Atrens A, Chen X, Xie Z, Sun D, Pan F (2019). Fabrication and characterization of an actively protective Mg-Al LDHs/Al2O3 composite coating on magnesium alloy AZ31. Appl. Surf. Sci..

[74] Duan H, Yan C, Wang F (2007). Effect of electrolyte additives on performance of plasma electrolytic oxidation films formed on magnesium alloy AZ91D. Electrochim. Acta.

[75] Lim TS, Ryu HS, Hong SH (2012). Electrochemical corrosion properties of CeO2−containing coatings on AZ31 magnesium alloys prepared by plasma electrolytic oxidation. Corros. Sci..

[76] Gnedenkov AS, Lamaka SV, Sinebryukhov SL, Mashtalyar DV, Egorkin VS, Imshinetskiy IM, Zavidnaya AG, Zheludkevich ML, Gnedenkov SV (2020). Electrochemical behaviour of the MA8 Mg alloy in minimum essential medium. Corros. Sci..

[77] Zhang S, Li Q, Chen B, Yang X (2010). Preparation and corrosion resistance studies of nanometric sol–gel-based CeO2 film with a chromium-free pretreatment on AZ91D magnesium alloy. Electrochim. Acta.

[78] Zhang G, Tang A, Wu L, Zhang Z, Liao H, Long Y, Li L, Atrens A, Pan F (2019). In-situ grown super- or hydrophobic Mg-Al layered double hydroxides films on the anodized magnesium alloy to improve corrosion properties. Surf. Coat. Technol..

